# Paralysis of the Accommodation of the Eye, Following an Attack of Diphtheria

**Published:** 1875-02-01

**Authors:** P. A. Callan

**Affiliations:** Assist. Surgeon New York Eye Infirmary


### PARALYSIS OF THE ACCOMMODATION OF THE EYE, FOLLOWING AN ATTACK OF DIPHTHERIA.

BY P. A. CALLAN, M.D., ASSIST. SURGEON NEW YORK EYE INFIRMARY.

THE two cases here presented, occurred in the service of Dr. Henry D. Noyes, at the N. Y. Eye and Ear Infirmary. Considering the large amount of diphtheria in the city at present, and the few cases of eye complications seen in the present epidemic, we think they may be of interest to the profession.

Mary L., age 11, Patterson, N. J. The patient, about the middle of September, had an attack of diphtheria, from which she apparently recovered, and she enjoyed perfect health up to the first of November, when she complained of not being able to see distinctly any object, being blurred and hazy, so that she was obliged to discontinue going to school. The mother of patient noticed about the same time, that she spoke through her nose, and quite indistinctly, having an impediment in her speech, as the mother designated it. This condition of affairs having remained unchanged, she was brought to the city, November 16, when she came under our observation for the first time. Patient's physique, fair; when spoken to, she readily responded, but her pronunciation was quite defective, and she spoke through her nose. Tongue and pharynx normal in appearance; the uvula quite long; and when patient was made to articulate, it moved very slightly. Patient had some difficulty in swallowing. The pupils of both eyes were slightly dilated; the vision of both eyes was $\frac{20}{70}$ with glasses + $\frac{1}{20}$ vision = $\frac{2}{20}$. At a distance of ten inches, she could not see ordinary print, and it was with difficulty, that she managed to make out Snellen 6½, at the ordinary reading distance, which should be read at 6½ feet, but with her proper correcting convex glass, she could read with comparative ease.

Ophthalmoscope showed a hypermetropic build of eye and a normal fundus. Patient had a hyperm etropic of $\frac{1}{20}$.

*Treatment.*—Tonics, with local applications of electricity. November 30, patient's vision has very much improved, but the voice remains much the same. Prognosis very good.

Joseph M., age 4½ years, N.Y. City. Patient had an attack of diphtheria about October 1st, from which he slowly recovered. About five weeks from his attack, the mother noticed that he could not see small objects which were in close proximity: small playthings for instance, would fall out of his hands and he could not find them—distant objects on the other hand were seen. At the same time, he had hemiplegia of the left side, with partial ptosis of the left eye.

Nov. 19.—The patient came under our observation for the first time, when we found paralysis of acc. in addition to the others just mentioned. Ophthalmoscopic examination showed the eye to be hypermetropic in build, and the fundus perfectly normal. Patient was treated as case No. 1.

Nov. 27.—Vision and ptosis much improved; hemiplegia much the same. —*N. Y. Med. Record.*

---

DR. MARY PUTNAM JACOBI, during the recent meeting of the New Yoik Pathological Society, temporarily occupied the chair during the presentation of a specimen by the President This is one way of answering the question of women's rights in medicine.—*N. Y. Med. Record.*

HINDRANCES TO VACCINATION.—It is of the first importance to uniform success of the practice of vaccination that the vaccinator shall be possessed of a special skill and knowledge of the subject. The physician must have acquired particular practical knowledge and an acquaintance, not only of the true genuine vaccination in all its regular stages, but he must be able to detect the least variation from the normal course at every stage. Vaccinators in Great Britain are required to stand an examination as to their qualifications before receiving an appointment. It is apprehended that great advantage would accrue to the people if the same rule were in existence in the United States.

It is the conviction of not only every medical man, but of every intelligent citizen, that a properly performed and successful vaccination, whether with humanized or animal virus, is as complete a protection against small-pox now as it ever was, and is a more perfect prophylactic than we possess against any other known disease. But it is of the highest importance, and a prerequisite to success, that the vaccinator obtain virus of unquestionable purity. The particular mode of introducing the virus perhaps makes but little difference in the result, provided no undue injury is done to the tissue, and the lymph, or dissolved crusts, are brought in direct contact with the absorbents, although the shape of the resulting vesicle may depend somewhat upon the particular operation. The thumb lancet is the best instrument to use.

Insusceptibility to vaccination is rare. Failure to induce vaccination, is more frequently the fault of the virus used, or of the operator. In reference to transmitting diseases in vaccine matter, Marson, who has vaccinated over fifty thousand individuals, "has never seen other diseases communicated with vaccine disease, nor does he believe in the popular reports that they are so communicated." Spurious vaccination has been found to pervert or greatly embarrass subsequent successful vaccination. Thus it is necessary to secure good and complete primary vaccination. It is probable that a fruitful cause of insufficient vaccination in the United States, originates from the want of quantity rather than defective quality of the vaccine fluid.—*J. M. Toner, M.D., American Public Health Association*, 1874.—*The Sanitarian.*

---

ADIPOCERE.—During the past summer, an example of the conversion of the tissues of the body into that spermaceti-like substance known as adipocere occurred in the body of a woman which had been dredged from the Thames, in London, after having been embedded in the mud for an unknown period—probably two or three years. Upon rapping, the body was hard and perfectly resonant, and the whole of the internal organs were converted into a solid mass, which, like the rest of the body, when cut into, had the appearance and consistence of hard, discolored wax. One leg was absent, having, probably, been separated by the weight of the mud when the body was pulled up by the dredger.—*N. Y. Med. Record.*

---

INFLUENCE OF A PROPER DRAINAGE AND WATER SUPPLY UPON THE MORTALITY OF A TOWN.—In the twelve years immediately preceding the completion of the drainage and water supply system of Salisbury, England, the yearly mortality amounted to 27 per 1,000. During the twelve years following, the mortality fell to 20 per 1,000; and during the last three years, it was only 17 per 1,000. During the latter period, typhoid fever was almost unknown, and the cholera, which in 1849, or before these changes had been made, was fatal in nearly 200 cases—was only fatal in 14 cases in 1854, the time at which the works were under completion. In 1866, there was not a single case in the town.—*Geigel's Offentl. Gesundheitspflege.*

